# Multimetal bioremediation from aqueous solution using dead biomass of *Mucor* sp. NRCC6 derived from detergent manufacturing effluent

**DOI:** 10.1007/s13353-023-00765-9

**Published:** 2023-07-05

**Authors:** Mervat Morsy Abass Ahmed El-Gendy, Shimaa M. Abdel-Moniem, Nabila S. Ammar, Ahmed Mohamed Ahmed El-Bondkly

**Affiliations:** 1 Chemistry of Natural and Microbial Products Department, National Research Centre, Dokki, Giza, 12622 Egypt; 2 Water Pollution Research Department, National Research Centre, El-Buhouth St., Dokki, Giza, 12622 Egypt; 3 Genetics and Cytology Department, National Research Centre, Dokki, Giza, 12622 Egypt

**Keywords:** Detergent industry effluents, Heavy metal adsorption, Optimization, Isotherm models, SEM-EDX, FTIR

## Abstract

Among ten metal-tolerant fungal isolates obtained from the microbiomes of detergent industry effluent, *Mucor* sp. NRCC6 showed the highest tolerance and an adaptive behavior toward the heavy metals Ni^2+^, Pb^2+^, Mn^2+^, and Zn^2+^. It gave the highest growth rates 0.790 ± 0.59, 0.832 ± 0.32, 0.774 ± 0.40, and 0.741 ± 1.06 mm/h along with the lowest growth inhibition 9.19, 4.37, 11.04, and 14.83% in the presence of Pb^2+^, Zn^2+^, Ni^2+^, and Mn^2+^, respectively, at a concentration of 5.0 g/L. Then, *Mucor* sp. NRCC6 was selected as a biotrap for the removal of these heavy metals. The optimized operating conditions were detected to be pH 6.0 for Pb^2+^, Zn^2+^, and Mn^2+^ and pH 5.5 for Ni^2+^ at 30 °C; agitation speed 150 rpm; contact time 30 min for Mn^2+^ and Ni^2+^, 30–60 min for Pb^2+^, and 90–180 min for Zn^2+^; NRCC6 biomass dosage 5.0 g/L for Ni^2+^ and Pb^2+^ and 10.0 g/L for Mn^2+^ and Zn^2+^; and initial concentration 12 mg/L of each ion in the multimetal aqueous solutions. Under these optimized conditions, the adsorption capacity for Pb^2+^, Ni^2+^, Mn^2+^, and Zn^2+^ reached 98.75, 59.25, 58.33, and 50.83%. The Langmuir isotherm was the best for describing the adsorption of Zn^2+^ (0.970) and Mn^2+^ (0.977). The Freundlich isotherm significantly giving a good fit to the adsorption of Pb^2+^ (0.998) while the adsorption of Ni^2+^ onto NRCC6 biomass can follow DKR (0.998). Furthermore, the current study revealed that *Mucor* sp. NRCC6 fungus is a new efficient and eco-friendly method that revealed a maximum removal of 100% for Pb^2+^ and Zn^2+^ as well as 97.39, 88.70, 78.95, 74.0, 70.22, 68.57, and 60.0% for Ni^2+^, Mn^2+^, Cd^2+^, Cu^2+^, Fe^3+^, As^2+^, and Cr^6+^ from the industrial wastewater, respectively.

## Introduction

The augmentation in industrial activity is important for the economic development of countries, but it causes environmental and health problems owing to its toxic effluents (Ahmed et al. 2021; Gül 2020). Globally, the detergent industries have been prioritized as one of the most polluting. Effluents from the manufacture of detergents contain high levels of oil and grease, biochemical oxygen demand (BOD), chemical oxygen demand (COD), suspended solids (SS), and a large number of heavy metals that can be toxic, reactive, carcinogenic, or ignitable to the aquatic flora and fauna (Akhtar and Mannan 2020; Jakovljević and Vrvić 2017). Consequently, without appropriate treatment and management strategies, the discharge of industrial wastewaters into water bodies can lead to horrific environmental and health impacts (Chettri et al. 2022; Mousavi and Khodadoost 2019). There are various methods of industrial wastewater treatment compared to them; mycoremediation can be a cost-effective, safe, and efficient method of substantial metals and pollutant decontamination (Alao and Adebayo 2022; Chugh et al. 2022; Jakovljević and Vrvić 2018). Heavy metals including Pb^2+^, Ni^2+^, Mn^2+^, and Zn^2+^ are non-biodegradable, tend to accumulate in the environment, enter the food chain, and pose potential threats to living organisms (Razzak et al. 2022). These heavy metals are known to be mutagens, carcinogenic, and impair the proper functioning of the kidneys, liver, spleen, heart, and reproductive systems (AL-Huqail and El-Bondkly 2022; El-Gendy et al. 2011). Moreover, the reactive oxygen species (ROS) produced by these heavy metals oxidize different important biomolecules such as nucleic acids, proteins, and lipids (El-Gendy and El-Bondkly 2016; Gheorghe et al. 2017).

Previous works proved that dead fungal biomass can be an efficient choice to reduce various toxic compounds and heavy metals from industrial wastewater with high adsorption capacity due to its large specific surface area and effective surface reactivity and can withstand temperature variation, microbial attack, and conditions of chemical instability (Paria et al. 2022; Ab Rhaman et al. 2022). Screening of industrial wastewater contaminated with heavy metals for the fungal microbiome has led to the classification of various indigenous fungi. For instant, Selvarajan et al. (2019) characterized the fungal diversity of various industrial effluents using a high-throughput sequencing method such as 6 phyla, 31 classes, 79 orders, 144 families, and 192 genera among which *Ascomycota* and *Basidiomycota* were the most dominant phyla with relative abundances of 38.31% and 33.51%, respectively. Moreover, several fungal species including *Trichoderma brevicompactum*, *Aspergillus ustus*, *Penicillium chrysogenum*, *Phanerochaete chrysosporium*, *Phlebia brevispora*, and *Phlebia floridensis* were isolated from various industrial effluents with high resistance to metals toxicity due to the adaptation to the environmental condition of the polluted sites and then the removal of heavy metals and other toxicants (El-Gendy et al. 2017a; Sharma et al. 2022).

Several works of literature have focused on the biotechnological importance of *Mucor* fungal biomass. *Mucor* species can raise and transform or break down hazardous compounds in a contaminated environment, as they have a highly versatile metabolic system that positions them as powerful microbial cell factories for various products including lipids, pigments, chitin/chitosan, polyphosphates, organic acids, and enzymes (Chai et al. 2019; Dzurendova et al. 2022; Hyde et al. 2020; Molaverdi et al. 2022). These fungal metabolites represent power and useful potential in bioremediation processes as effective bioadsorbents (Lima et al. 2020; Nguyen et al. 2020).

Thus, in this study, the hyper multimetal-tolerant strain *Mucor* sp. NRCC6 isolated from the microbiome of the detergent industry effluent has been explored for its multisorption efficiency and adsorption capacity of Mn^2+^, Zn^2+^, Ni^2+^, and Pb^2+^ in their co-presence in the synthetic multimetal solution and industrial wastewaters. Furthermore, the batch adsorption conditions including pH, contact time, biomass dosage, and initial metal concentration were optimized under a multimetal system. Moreover, isothermal models (Langmuir, Freundlich, and Dubinin–Kaganer–Radushkevich) were evaluated under optimized conditions. Finally, the effect of Pb^2+^, Ni^2+^, Mn^2+^, and Zn^2+^ blind on NRCC6 mycelial surface morphology was assessed by SEM, EDX, and FTIR spectroscopy.

## Materials and methods

### Metal ion solutions and factory effluent preparation

A stock of multimetal ion solution composed of Pb^2+^ (Pb (CH_3_COO)_2_), Ni^2+^ (NiCl_2_.6H_2_O), Mn^2+^ (MnSO_4_·H_2_O), and Zn^2+^ [Zn(CH_3_CO_2_)_2_] was prepared by dissolving a proper amount of each metal in deionized water to prepare a concentration of 1000 mg/L. The desired concentrations of C_o_ = 12, 25, 50, and 100 mg/L of each ion in the multimetal solution were gotten by successive dilution of the standard stock solution with appropriate amounts of deionized water. The medium pH was adjusted from 2.0 to 6.0 using 0.1 M HCl and 0.1 M NaOH solutions. Real wastewater samples belonging to the detergent industry were collected from the drainage areas of detergent industry wastewater at the Industrial Zone, 10th of Ramadan, Sharkia, Egypt, in polyethylene bottles at 1 L. The collected wastewater samples were gathered and divided into three portions. The first was processed immediately for the isolation of their fungal mycobiome; the second was placed in sterile 250 mL conical flasks containing 2.5 mL nitric acid (conc.) and kept at 4 °C until analyses for their characteristics before treatment within 24 h of collection by using Agilent 5100 Synchronous Vertical Dual View (SVDV) ICP-OES, with Agilent Vapor Generation Accessory VGA 77. The third was kept at 80 °C until treatment with the dead biomass of selected fungus based on the optimization experiments followed by analyses for its Pb^2+^, Ni^2+^, Mn^2+^, and Zn^2+^ contents and other characters.

### Isolation of mycobiome from the detergent industrial effluents

The fungal biosorbents were isolated from detergent manufacturing industrial wastewater (drainage areas, Industrial Zone, 10th of Ramadan, Sharkia, Egypt). Detergent effluent samples were filtered, serially diluted using the serial dilution technique, and inoculated into a potato dextrose agar (PDA) medium. The plates were incubated for 10 days at 28 °C, and isolates fulfilling all fungal growth criteria were transferred periodically to the PDA medium. The hyper multimetal-tolerant isolate NRCC6 was selected, identified, and analyzed for its biosorption efficiency and adsorption capacity of different heavy metals from the multimetal aqueous solutions as previously described. Moreover, the ability of this strain to enhance the industrial wastewater properties was evaluated under the conditions optimized earlier in the batch process including pH, contact time, biomass dosages, and initial heavy metal concentrations.

### Determination of fungal isolates resistant to heavy metals by growth rate and inhibition

In this study, we decided to test and screen the obtained fungal microbiome according to its tolerance to heavy metals such as Zn^2+^, Pb^2+^, Ni^2+^, and Mn^2+^. To assess the tolerance of fungal isolates, a 7 mm diameter mycelial disc from 3-day-old culture of each fungus grown in PDA without metals was inoculated aseptically and individually into the middle of Petri dishes containing one of the metal ions under study at different concentrations of 0.15, 0.25, 0.50, 1.0, 2.0, 3.0, 4.0, and 5.0 g/L in PDA medium, individually along with control and incubated for 5 days at 25 °C. The average growth diameter was measured with a ruler daily to determine metal tolerance, and the end of the trial was considered when the fungal growth in the medium without metals (control) filled a Petri dish (3 to 5 days). The radial growth of the mycelia was measured daily in triplicate, the averages were plotted, and the slope was applied to determine the growth rate in mm per hour. To determine the percentage of growth inhibition, the average growth of the mycelial mat (mm) was measured, taking the growth diameter of the control fungus as 100% and then subtracting it from the growth percentage of the fungus that was inhibited. The selected hyper multimetal-tolerant isolate NRCC6 has been subjected to various studies and evaluations.

### Morphological and molecular identification of the hyper tolerant isolate NRCC6

#### Macroscopic and microscopic features

Macroscopic characteristics of the fungal isolate were determined using PDA, malt extract agar (MEA) (Difco Laboratories, USA), and Czapek yeast autolysate agar (CYA agar). Plates were incubated in the dark at 25 °C for 5 days to study its colonial features. Colony features such as growth, obverse-reverse colony color, and change in colony color with time were examined. Micromorphological characteristics such as sporangiophores, sporangium, columellae, and chlamydospore formation were carried out by growing the isolates on MEA that incubated at 25 °C and staining slides of fresh 7-day-old culture to observe under the microscope. Fifty measurements were prepared for each microscopic character, and the averages were calculated. The optimal growth and sporulation temperature of strains were determined on MEA at 5, 10, 15, 16, 18, 20, 25, 30, 32, 35, and 37 °C, respectively. Furthermore, the ability to grow as yeast was tested by inoculating 10^5^ spores/mL from 3–5-day-old colonies on MEA onto 100 mL Erlenmeyer flasks containing 50 mL yeast-peptone-glucose broth (YPG: 0.3% yeast extract, 1% peptone, 3% glucose) and incubated under the static condition at 25 °C for 48 h; then, morphology was studied microscopically. Phenotypic characteristics were studied using the previous taxonomic keys (De Hoog et al. 2000, 2015; Hibbett et al. 2007; Rayner 1970; Rippon 1988; Schipper 1976, 1978; Schipper and Samson 1994; Vellanki et al. 2018; Watanabe 2009).

#### Molecular identification

Fungal isolate NRCC6 was grown on PDA for 5 days at 28 °C. Genomic DNA was extracted from pure cultures. The presence of DNA was established on a 0.8% agarose gel stained with ethidium bromide. DNA of the isolate served as the template for the PCR amplification, and for isolate NRCC6, a partial sequence of the rDNA with primers ITS1 and ITS4 was amplified for the internal transcribed spacer (ITS) region of rDNA, amplification, PCR product purification, and sequencing in both directions that were performed following the previous protocol (El-Bondkly 2012; El-Bondkly and El-Gendy 2022; El-Gendy et al. 2018; Hibbett et al. 2007; Machouart et al. 2006; White et al. 1990). The software SeqMan (DNAStarLasergene) was applied to edit, assemble, and get the consensus sequences, which were then deposited in GenBank of the National Center for Biotechnology Information (NCBI). The sequence obtained was compared with other fungal sequences deposited in the NCBI databases. Alignment of those sequences and the phylogenetic analysis for each locus were achieved with the MEGA11. Phylogenetic reconstruction was made with the phylogenetic marker (ITS) recommended for an accurate identification at the species level using maximum likelihood (ML) analyses, with the MEGA11 software (Kumar et al. 2018; Tamura et al. 2011, 2021).

### Preparation of the dead fungal biomass (adsorbent)

Ten-day-old NRCC6 culture spores (10^6^ spore/mL) were transferred individually into 500 mL Erlenmeyer flasks containing 100 mL potato dextrose broth (PDB) medium and incubated at 25 °C and 150 rpm for 5 days on a rotary shaker. The resultant biomass was pelletized by filtration through filter papers (Whatman No. 1), washed five times with 0.1 M NaCl, followed by deionized water to remove non-biomass particles, then autoclaved, washed with 0.1 M NaCl, transferred to pre-weighted aluminum foil caps, and dried in an oven at 60 °C until a constant weight was obtained. The dead biomass was pulverized to a fine powder using a porcelain mortar and preserved in sterile polyethylene bottles at 4 °C until use.

### Evaluation of adsorption performance of the selected fungal isolate NRCC6

Unless stated otherwise, the biosorption tests were conducted under the multimetal system in quick-fit flasks containing a biosorbent dosage of 1 g/L of the dead biomass of NRCC6 strain in a working volume of 50 mL aliquots of a mixture of Pb^2+^, Ni^2+^, Mn^2+^, and Zn^2+^ at a concentration of 100 mg/L (25 mg/L for each). Flasks were kept on rotary shakers (150 rpm) at 30 °C and pH 5.5 for 30 min. The supernatants were analyzed for residual heavy metals. Accuracy and precision of the metal ion measurements were established using external reference standards from Merck and standard reference material and quality control sample from the National Institute of Standards and Technology (NIST) to confirm the instrument reading (Li et al. 2009; Singh et al. 2007). Metal solutions without biomass were served as control, trials were conducted in triplicate, and average values were computed. The metal removal efficiency in terms of percentage (*R*%) was determined in the multimetal solution of Pb^2+^, Ni^2+^, Mn^2+^, and Zn^2+^ or in real wastewater:

1 $$R=\frac{C_o-{C}_f}{C_o}\times 100\%$$

where *R* is the biosorption efficiency percentage (%) of each metal separately; *C*_*o*_ is the initial metal concentration (mg/L), and *C*_*f*_ is the equilibrium or final concentration for each metal calculated separately. Moreover, the adsorption capacity of fungal biomass was estimated by the equation:

2 $${q}_t=\left({C}_o-{C}_t\right)\times \frac{V}{m}$$

where *q*_*t*_ is adsorption capacity (mg/g), *C*_*t*_ is the metal concentration (mg/L) at time = *t* (min), *V* is the solution volume (L), and *m* is adsorbent mass (g).

### Optimization of the biosorption batch factors

Batch experiments were conducted under the coexistence of Pb^2+^, Ni^2+^, Mn^2+^, and Zn^2+^ solutes in 250-mL Erlenmeyer flasks, following optimization of a one-factor-at-a-time technique. In the first trial, the impact of pH on metal biosorption by NRCC6 biomass was evaluated by varying adjusted pH values 2, 4, 5.5, and 6 using dilute HCl or NaOH; the effect of the adsorption time was evaluated at 10, 20, 30, 60, 90, and 180 min at the optimum pH. Moreover, the effects of the dead fungal biomass dosages (1, 2, 5, and 10 mg/mL) were evaluated at the optimum pH and contact time. The initial concentration of Pb^2+^, Ni^2+^, Mn^2+^, and Zn^2+^ metals (*C*_*o*_ = 12, 25, 50, and 100 mg/L of each metal in the mixture) was evaluated at the proceeding optimum conditions. In each experiment, flasks were allowed to attain equilibrium on the rotary shaker, and samples were collected after the appropriate time; the aqueous solutions were filtered, and each supernatant was analyzed for residual concentration of each metal and its bioremoval efficiency (%) and adsorption capacity (mg/g) to determine the optimum process parameters.

### Adsorption isotherm

The adsorption of heavy metal ions by the fungal biomass was evaluated by various adsorption isotherm models including Langmuir, Freundlich, and Dubinin–Kaganer–Radushkevich (DKR) models.

#### Langmuir isotherm model

Langmuir isotherm equation is represented by the following equation:

3 $$\frac{C_e}{Q_e}=\frac{1}{Q_{\max {K}_1}}+\frac{C_e}{Q_{\textrm{max}}}$$

where *Q*_*e*_ is the equilibrium adsorption capacity (mg/g), *C*_*e*_ is the concentration of adsorbate molecule remaining in solution at equilibrium (mg/L), *Q*_max_ is the adsorption of maximum ions per unit mass of fungi, (mg/g) related to adsorption capacity that represents monolayer coverage, and *K*_*L*_ is the Langmuir constant equivalent to the enthalpy of adsorption (L/mg). Therefore, the linear plot of *C*_*e*_/*qe* versus *Ce* gives a straight line of slope 1/*q*_max_ and intercepts 1/(*q*_max_K_L_) (Dąbrowski 2001). The Langmuir model parameters were applied to calculate the separation factor RL, as expressed by Eq. (4) according to Fawzy et al. (2018).

4 $$\textrm{RL}=\frac{1}{1+{K}_l{C}_o}$$

#### Freundlich isotherm model

This model suggests the heterogeneous adsorption of the surface that has unequal available sites. The linear Freundlich isotherm equation can be written as follows:

5 $$\ln {Q}_e=\ln {K}_f+\frac{1}{n}\ln {C}_e.$$

where *Q*_*e*_ (mg/g) is the amount of metal ion adsorbed on adsorbent at equilibrium, *C*_*e*_ (mg/L) is the equilibrium concentration of a metal ion in the solution, *K*_*f*_ (mg^1−1/*n*^ L^1/*n*^ g^−1^) is a Freundlich isotherm constant describing the adsorption capacity and is an empirical parameter related with multiple layer coverage (Ayawei et al. 2017; Hamdaoui and Naffrechoux 2007).

#### Dubinin–Radushkevich (DKR) isotherm

The results were also fitted with the DKR isotherm model to estimate the nature of the adsorption process as chemical or physical and evaluate the mean energy of sorption. The linear equation of the DKR isotherm is

6 $${q}_{e={q}_m{\exp}^{-}}\beta \varepsilon 2$$

where *q*_*e*_ is the number of metal ions adsorbed per unit weight of adsorbent (mol/g), *q*_*m*_ is the maximum sorption capacity, *b* is the activity coefficient related to mean sorption energy, and Ɛ is the Polanyi potential, which is equal to

7 $$\varepsilon = RTln\ \left(1+{~}^{\Delta \mathrm P}\!\left/ \!{~}_{\mathrm L}\right.\right)$$

where *R* is the gas constant (kJ/kmol K) and *T* is the temperature (K). By plotting a relationship between ln*q*_*e*_ and ε^2^, β and *q*_DR_ can be obtained. (D-R) isotherm parameter β is applied to determine adsorption energy E (KJ/mol) as follows:

$$E=\frac{1}{\sqrt{-2\beta }}$$(8).

### Scanning electron microscope (SEM), energy-dispersive X-ray spectroscopy (EDX), and Fourier transform infrared spectroscopy (FTIR) analyses

EDX linked to SEM was applied to determine the chemical characterization of fungal biomass NRCC6 strain before and after adsorption of heavy metal ions Pb^2+^, Zn^2+^, Ni^2+^, and Mn^2+^. The fungal biomass amended with a multimetal solution of these ions at the initial concentration of 100 mg/L of each was used for SEM analysis (SEM Quanta FEG 250 with field emission gun, FEI Company—Netherlands) at the Central Laboratory (National Research Centre, Egypt). Confirmation of the presence of metal ions on the fungal biomass surface was tested using EDX analysis (X-ray micro-analyzer connected to a scanning electron microscope). The individual ratios given represent the average of ten measurements. The functional groups on the biomass surface were defined by a Fourier transform infrared spectrometer (Bruker Vertex80v, Germany). In FTIR analysis, pressed potassium bromide (KBr) pellets were applied to measure the transmittance spectra recorded in the range of 4000–400 cm^−1^ with a resolution of 4 cm^−1^ at the Central Laboratory of National Research Centre, Egypt, to define the vibration frequency groups in the biosorbent NRCC6 before and after biosorption of heavy metals under the study from the multimetal solution. The obtained spectral data were compared with the reference chart to identify the functional groups present in the sample.

### Characterization of industrial wastewater

Samples collected from the detergent industry effluents at the Industrial Zone, 10th of Ramadan, Sharkia, Egypt, were subjected to centrifugation at 2000 rpm for 2 min, filtration by Whatman filter paper with 0.2 μm pore size, acid digestion according to APHA (2017), and followed by determination of the initial and final concentrations of metal ions before and after treatment with NRCC6. Moreover, the other wastewater parameters include pH, temperature, turbidity, total suspended solids (TSS), total dissolved solids (TDS), oil and grease, chemical oxygen demand (COD), biological oxygen demand (BOD), dissolved oxygen (DO), PO4^3−^, SO_4_^2−^, NO_3_^1−^, and NH_4_–N that were analyzed following the standard methods for the examination of water and wastewater (APHA 2017).

### Statistical analysis

The results were statistically processed by the analyses of variance (ANOVA), followed by T- or Tukey’s tests when significant effects were detected (*P* ≤0.05). Data were expressed as means ± standard error.

## Results and discussions

### Screening and selection of the high metals tolerance fungal strains

The tolerant of the fungal mycobiota obtained from the detergents industrial effluents against Ni^2+^, Pb^2+^, Mn^2+^, and Zn^2+^ was evaluated to select the highly tolerant strain that showed the highest growth rates along with the lowest growth reduction in the presence of each heavy metal in the PDA growth medium. Data in Table 1 indicated that all ten fungal isolates were able to grow in the presence of Ni^2+^, Pb^2+^, Mn^2+^, or Zn^2+^ at concentrations ranging from 0.15 to 5.0 g/L in the growth medium. At the highest concentration (5 g/L), the fungal isolate NRCC6 followed by NRCC5 and NRCC10 showed the highest growth rates (GR; 0.790 ± 0.59, 0.567 ± 0.42, and 0.552 ± 0.34 mm/h) along with the lowest growth inhibition (GI; 9.19, 21.90, and 36.19%) against Pb^2+^, respectively, while NRCC6, NRCC5, and NRCC1 isolates showed the highest tolerant against Zn^2+^ with growth rate equal to 0.832 ± 0.82, 0.530 ± 0.67, and 0.350 ± 0.24 mm/h while their growth was repressed by 4.37, 26.99, and 27.39%, respectively, at 5.0 g/L of Zn^2+^ concentration. However, isolates NRCC6 and NRCC10 (GR; 0.774 ± 0.40 and 0.783 ± 0.36 mm/h with GI; 11.04 and 9.48%) showed the highest adaptation toward 5 g/L of Ni^2+^, respectively, but isolates NRCC6 and NRCC5 were more adaptive for manganese at 5.0 g/L (GR; 0.741 ± 1.06 and 0.532 ± 0.95 mm/h along with GI; 14.83 and 26.72%, respectively) in the growth medium (Table 1). *Compared to the control, the growth rate of* NRCC6*, 0.870* ± 0.85 mm/h, *was* unaffected *in the presence of Zn*^*2+*^ *up to 2.0 g/L and then slightly decreased* by 1.04, 2.64, and 4.37% at 3, 4, and 5 g/L of Zn^2+^, respectively, while its growth rates were repressed by 1.26 to 9.19%, *3.56* to *11.04*%*,* and *6.89* to *14.83*% *with concentrations ranging from 0.15 to 5.0 g/L of Pb*^2+^*, Ni*^2+^*, and Mn*^2+^*, respectively (*Table 1*).* Then, the fungal strain under the isolation code NRCC6, which showed the hyper metal tolerance and adaptive behavior against Ni^2+^, Pb^2+^, Mn^2+^, and Zn^2+^, was selected for further studies. In line with our results, Girdhar et al. (2022) reported that fungi are useful in maintaining tolerance against heavy metals in different polluted sites by developing different resistance methods against heavy metals to survive through adaptation or mutation, and they can reduce heavy metals from the environment to some extent. Mycoremediation using the indigenous fungal microbiomes of contaminated sites including *Aspergillus fumigatus*, *Aspergillus niger*, *Aspergillus terreus*, *Macrophomina phaseolinia*, *Penicillium* sp., *Rhizopus stolonifer*, *Trichoderma viride*, and *Trichoderma longibrachiatum* could be alternative biotechnology for the removal of toxic metals (El-Gendy et al. 2011, 2017a; Alzahrani and El-Gendy 2019). Moreover, Hoque and Fritscher (2019) stated that *Mucor hiemalis* EH8 and EH11 isolated from the microbiome of cold sulfidic spring waters showed extraordinary biosorption and uptake capacity of metals in the co-presence of harmful multimetals and organic toxins because they possess an additional repertoire of enzymes for the detoxification of heavy metals and organic toxins.

**Table 1:** Growth rates and growth percentage inhibition values for fungal isolates derived from the detergent industrial effluents cultivated in PDA supplemented with different metal ion concentrations

Isolate and heavy metal concentration	Heavy metal*							
	Pb^2+^		Zn^2+^		Ni^2+^		Mn^2+^	
	Growth rate (mm/h)	Growth inhibition (%)	Growth rate (mm/h)	Growth inhibition (%)	Growth rate (mm/h)	Growth inhibition (%)	Growth rate (mm/h)	Growth inhibition (%)
0.15 g/L								
NRCC1	0.394 ± 0.24	18.26	0.482 ± 0.43	0.00	0.362 ± 0.05	24.89	0.482 ± 1.24	0.00
NRCC2	0.425 ± 0.41	18.27	0.520 ± 0.56	0.00	0.461 ± 0.11	11.35	0.489 ± 1.16	5.96
NRCC3	0.680 ± 0.33	0.00	0.490 ± 0.50	27.94	0.518 ± 0.15	23.82	0.490 ± 1.05	27.94
NRCC4	0.530 ± 0.38	0.00	0.478 ± 0.45	9.81	0.467 ± 0.12	11.89	0.520 ± 1.18	1.89
NRCC5	0.726 ± 0.41	0.00	0.710 ± 0.90	2.20	0.658 ± 0.26	9.37	0.690 ± 1.30	4.96
NRCC6	0.859 ± 0.46	1.26	0.870 ± 0.85	0.00	0.839 ± 0.35	3.56	0.810 ± 1.71	6.89
NRCC7	0.289 ± 0.21	2.37	0.275 ± 0.26	7.09	0.295 ± 0.01	0.34	0.296 ± 0.64	0.00
NRCC8	0.333 ± 0.29	11.67	0.377 ± 0.38	0.00	0.330 ± 0.05	12.47	0.377 ± 0.78	0.00
NRCC9	0.450 ± 0.32	7.41	0.486 ± 0.50	0.00	0.450 ± 0.17	7.41	0.471 ± 1.04	3.09
NRCC10	0.828 ± 0.54	4.28	0.632 ± 0.54	26.94	0.865 ± 0.42	0.00	0.690 ± 1.24	20.23
0.25 g/L								
NRCC1	0.390 ± 0.35	19.09	0.470 ± 0.44	2.49	0.360 ± 0.04	25.31	0.480 ± 1.24	0.42
NRCC2	0.416 ± 0.37	20.00	0.511 ± 0.61	1.73	0.449 ± 0.14	13.65	0.470 ± 1.09	9.62
NRCC3	0.624 ± 0.44	8.24	0.490 ± 0.57	27.94	0.506 ± 0.19	25.59	0.455 ± 1.03	33.09
NRCC4	0.524 ± 0.38	1.13	0.470 ± 0.39	11.32	0.451 ± 0.13	14.91	0.500 ± 1.18	5.66
NRCC5	0.700 ± 0.61	3.58	0.684 ± 0.70	5.79	0.620 ± 0.24	14.60	0.662 ± 1.23	8.82
NRCC6	0.859 ± 0.69	1.26	0.870 ± 0.90	0.00	0.839 ± 0.36	3.56	0.806 ± 1.79	7.36
NRCC7	0.269 ± 0.24	9.12	0.250 ± 0.18	15.54	0.287 ± 0.05	3.04	0.296 ± 0.62	0.00
NRCC8	0.325 ± 0.37	13.79	0.363 ± 0.27	3.71	0.300 ± 0.07	20.42	0.370 ± 0.74	1.86
NRCC9	0.438 ± 0.42	9.88	0.476 ± 0.49	2.06	0.400 ± 0.13	17.97	0.450 ± 0.95	7.41
NRCC10	0.800 ± 0.70	7.52	0.519 ± 0.51	40.00	0.865 ± 0.41	0.00	0.671 ± 1.18	22.43
0.5 g/L								
NRCC1	0.374 ± 0.32	22.41	0.450 ± 0.44	6.64	0.342 ± 0.0	29.05	0.461 ± 1.19	4.36
NRCC2	0.400 ± 0.37	23.08	0.503 ± 0.50	9.62	0.437 ± 0.05	15.96	0.451 ± 1.23	13.27
NRCC3	0.600 ± 0.54	11.77	0.477 ± 0.46	29.86	0.486 ± 0.12	28.53	0.450 ± 1.17	33.82
NRCC4	0.520 ± 0.48	1.89	0.458 ± 0.49	13.59	0.439 ± 0.07	17.17	0.481 ± 1.28	9.25
NRCC5	0.690 ± 0.60	4.96	0.670 ± 0.75	7.71	0.620 ± 0.21	14.60	0.650 ± 1.30	10.47
NRCC6	0.859 ± 0.76	1.26	0.870 ± 0.90	0.00	0.839 ± 0.36	3.56	0.806 ± 1.76	7.36
NRCC7	0.250 ± 0.23	15.54	0.238 ± 0.30	19.59	0.270 ± 0.0	8.78	0.296 ± 0.62	0.00
NRCC8	0.315 ± 0.37	16.45	0.360 ± 0.42	4.51	0.288 ± 0.06	23.61	0.361 ± 0.74	4.24
NRCC9	0.429 ± 0.35	11.73	0.460 ± 0.51	5.35	0.390 ± 0.01	19.75	0.433 ± 0.85	10.91
NRCC10	0.797 ± 0.75	7.86	0.462 ± 0.49	46.59	0.865 ± 0.35	0.00	0.612 ± 1.14	29.25
1.0 g/L								
NRCC1	0.351 ± 0.33	27.18	0.437 ± 0.48	9.34	0.329 ± 0.04	31.74	0.436 ± 1.24	9.54
NRCC2	0.379 ± 0.37	27.12	0.484 ± 0.56	6.92	0.419 ± 0.10	19,42	0.430 ± 1.16	17.31
NRCC3	0.558 ± 0.58	17.94	0.461 ± 0.50	32.21	0.450 ± 0.19	33.82	0.423 ± 1.01	37.79
NRCC4	0.508 ± 0.63	4.15	0.450 ± 0.43	15.09	0.420 ± 0.14	20.76	0.473 ± 1.32	10.76
NRCC5	0.669 ± 0.45	7.85	0.650 ± 0.68	10.46	0.600 ± 0.24	17.36	0.638 ± 1.64	12.12
NRCC6	0.859 ± 0.74	1.26	0.870 ± 0.90	0.00	0.827 ± 0.31	4.94	0.790 ± 1.82	9.19
NRCC7	0.231 ± 0.21	21.96	0.226 ± 0.28	23.65	0.240 ± 0.0	18.92	0.292 ± 0.51	1.35
NRCC8	0.301 ± 0.28	20.16	0.347 ± 0.36	7.96	0.262 ± 0.07	30.50	0.340 ± 0.64	9.81
NRCC9	0.415 ± 0.37	14.61	0.443 ± 0.43	8.85	0.366 ± 0.13	24.69	0.415 ± 0.77	14.61
NRCC10	0.780 ± 0.68	9.83	0.421 ± 0.39	51.33	0.865 ± 0.34	0.00	0.547 ± 0.84	36.76
2.0 g/L								
NRCC1	0.320 ± 0.25	33.61	0.435 ± 0.44	9.75	0.304 ± 0.02	36.93	0.420 ± 1.19	12.86
NRCC2	0.363 ± 0.27	30.19	0.470 ± 0.50	9.62	0.400 ± 0.07	23.08	0.417 ± 1.05	19.81
NRCC3	0.540 ± 0.48	20.59	0.440 ± 0.43	35.29	0.428 ± 0.11	37.06	0.403 ± 1.02	40.74
NRCC4	0.494 ± 0.43	6.79	0.439 ± 0.43	17.17	0.410 ± 0.07	22.64	0.460 ± 1.15	13.21
NRCC5	0.650 ± 0.56	10.46	0.633 ± 0.70	12.81	0.576 ± 0.14	20.66	0.618 ± 1.60	14.88
NRCC6	0.840 ± 0.66	3.45	0.870 ± 0.90	0.00	0.819 ± 0.29	5.86	0.782 ± 1.78	10.12
NRCC7	0.219 ± 0.22	26.01	0.237 ± 0.22	19.93	0.220 ± 0.05	25.68	0.280 ± 0.50	5.41
NRCC8	0.287 ± 0.27	23.87	0.334 ± 0.30	11.41	0.240 ± 0.06	36.34	0.320 ± 0.64	15.12
NRCC9	0.400 ± 0.42	17.69	0.437 ± 0.55	10.08	0.352 ± 0.13	27.57	0.400 ± 0.69	17.69
NRCC10	0.754 ± 0.60	12.83	0.389 ± 0.41	55.03	0.849 ± 0.40	1.85	0.500 ± 0.78	42.19
3.0 g/L								
NRCC1	0.300 ± 0.39	37.76	0.403 ± 0.51	16.39	0.290 ± 0.0	39.83	0.392 ± 0.73	18.67
NRCC2	0.336 ± 0.34	35.39	0.449 ± 0.47	13.65	0.381 ± 0.04	26.73	0.400 ± 0.86	23.08
NRCC3	0.500 ± 0.54	26.47	0.413 ± 0.56	39.27	0.400 ± 0.09	41.18	0.384 ± 0.65	43.53
NRCC4	0.481 ± 0.48	9.25	0.416 ± 0.53	21.51	0.384 ± 0.06	27.55	0.455 ± 0.92	14.15
NRCC5	0.629 ± 0.59	13.36	0.615 ± 0. 70	15.15	0.556 ± 0.20	23.42	0.600 ± 1.00	17.36
NRCC6	0.827 ± 0.80	4.94	0.861 ± 0.91	1.04	0.808 ± 0.34	7.13	0.771 ± 1.19	11.38
NRCC7	0.194 ± 0.14	34.46	0.221 ± 0.32	25.39	0.200 ± 0.01	32.43	0.277 ± 0.62	6.42
NRCC8	0.265 ± 0.29	29.71	0.328 ± 0.41	12.99	0.215 ± 0.03	42.97	0.288 ± 0.74	23.61
NRCC9	0.361 ± 0.42	25.72	0.398 ± 0.50	18.11	0.329 ± 0.09	32.31	0.350 ± 0.84	27.98
NRCC10	0.680 ± 0.63	21.39	0.327 ± 0.41	62.19	0.830 ± 0.45	4.05	0.448 ± 0.90	48.21
4.0 g/L								
NRCC1	0.271 ± 0.30	43.78	0.378 ± 0.34	21.58	0.255 ± 0.04	47.09	0.342 ± 0.62	29.05
NRCC2	0.300 ± 0.37	42.32	0.404 ± 0.40	22.31	0.331 ± 0.09	36.35	0.360 ± 0.80	30.77
NRCC3	0.450 ± 0.44	33.82	0.400 ± 0.45	41.18	0.372 ± 0.16	45.29	0.361 ± 0.69	46.91
NRCC4	0.469 ± 0.38	11.51	0.383 ± 0.36	27.74	0.350 ± 0.11	33.96	0.433 ± 0.87	18.30
NRCC5	0.595 ± 0.45	18.04	0.590 ± 0.56	18.73	0.517 ± 0.22	28.79	0.590 ± 1.05	18.73
NRCC6	0.812 ± 0.66	6.67	0.847 ± 0.88	2.64	0.790 ± 0.29	9.19	0.759 ± 1.13	12.76
NRCC7	0.170 ± 0.19	42.57	0.191 ± 0.12	35.47	0.172 ± 0.00	41.89	0.269 ± 0.60	9.12
NRCC8	0.200 ± 0.22	46.95	0.265 ± 0.26	29.71	0.184 ± 0.04	51.19	0.258 ± 0.68	31.57
NRCC9	0.300 ± 0.32	38.27	0.365 ± 0.40	24.89	0.280 ± 0.08	42.39	0.300 ± 0.73	38.27
NRCC10	0.626 ± 0.50	27.63	0.273 ± 0.21	68.44	0.810 ± 0.40	5.43	0.405 ± 0.84	53.18
5.0 g/L								
NRCC1	0.240 ± 0.15	50.21	0.350 ± 0.24	27.39	0.222 ± 0.10	53.94	0.317 ± 0.57	34.23
NRCC2	0.259 ± 0.19	50.19	0.346 ± 0.30	33.46	0.283 ± 0.15	45.58	0.328 ± 0.76	36.92
NRCC3	0.400 ± 0.37	41.18	0.346 ± 0.45	49.12	0.329 ± 0.19	51.62	0.302 ± 0.59	55.59
NRCC4	0.329 ± 0.39	37.93	0.340 ± 0.33	35.85	0.313 ± 0.23	40.94	0.301 ± 0.69	43.21
NRCC5	0.567 ± 0.42	21.90	0.530 ± 0.67	26.99	0.500 ± 0.24	31.13	0.532 ± 0.95	26.72
NRCC6	0.790 ± 0.59	9.19	0.832 ± 0.82	4.37	0.774 ± 0.40	11.04	0.741 ± 1.06	14.83
NRCC7	0.161 ± 0.11	45.61	0.180 ± 0.19	39.19	0.159 ± 0.00	46.28	0.213 ± 0.51	28.04
NRCC8	0.140 ± 0.07	62.87	0.260 ± 0.22	31.04	0.150 ± 0.00	60.21	0.200 ± 0.56	46.95
NRCC9	0.276 ± 0.19	43.21	0.319 ± 0.30	34.36	0.225 ± 0.13	53.70	0.240 ± 0.62	50.62
NRCC10	0.552 ± 0.34	36.19	0.210 ± 0.21	75.72	0.783 ± 0.36	9.48	0.346 ± 0.74	60.00

*All treatments were tested in triplicate. Values showed the average growth rate (mm/h), growth inhibition (%), and ± standard deviation

### Characterization and identification of the high heavy metals tolerance isolate NRCC6

#### Morphological description

**Fig. 1:**
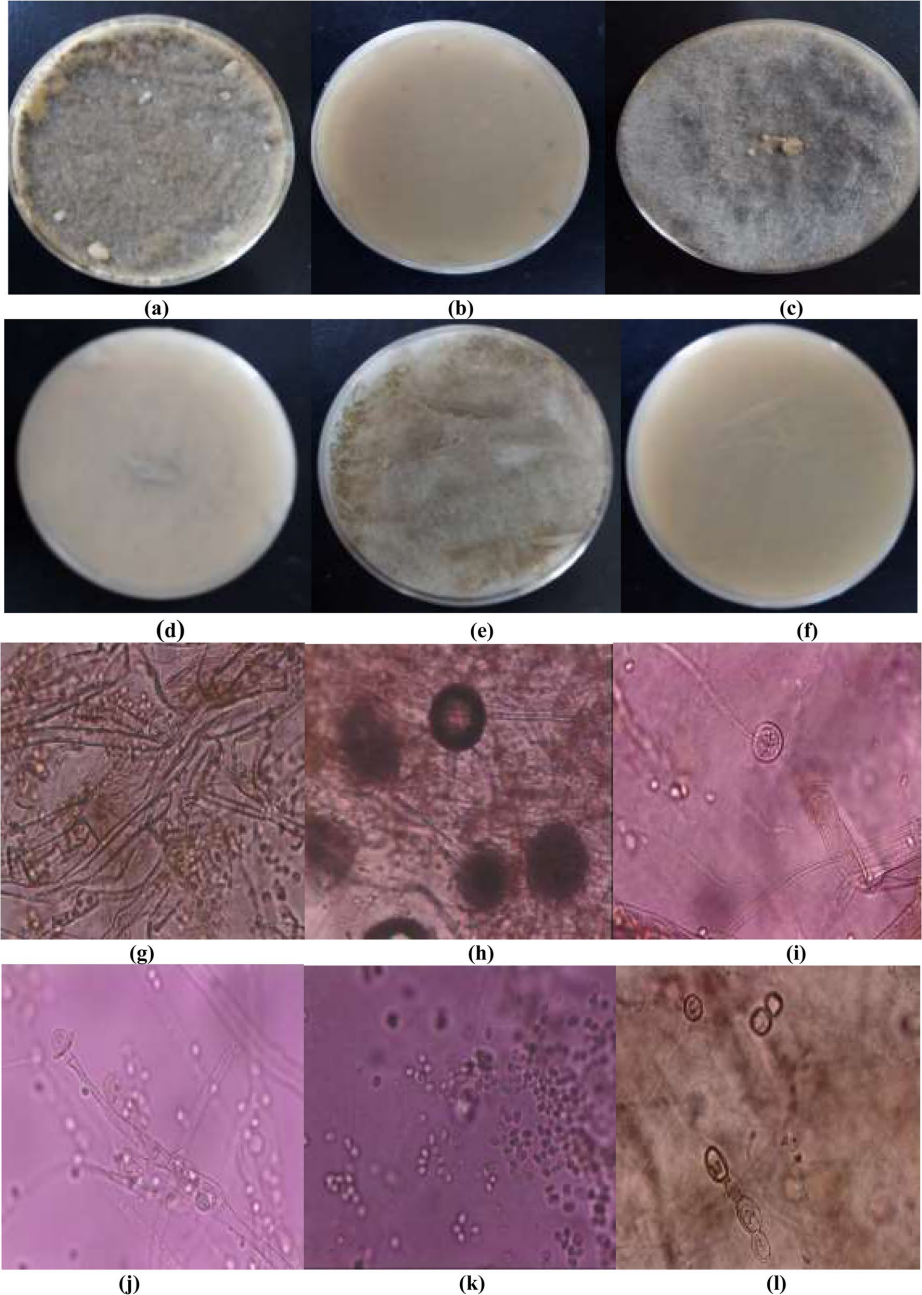
**a**–**f** Front and reverse of 3-day-old culture on MEA, PDA, and CYA; **g**, **h** sporangiophore with sporangium and columella; **i** columella; **j** bursting of sporangia to release sporangiospores; **k** sporangiospores; **l** chlamydospores at the later stage of growth of the metal-tolerant fungal isolate NRCC6

Colonies of NRCC6 strain on MEA are 62 mm in diameter after 3 days of incubation at 25 °C, completely covering a Petri plate (90 mm) by the fifth day, floccose, cottony, and ash gray with a light grayish brown reverse (Fig. 1a, b). The NRCC6 colonies on the PDA are gray cotton candy, very fast growing, and rapidly filling the entire Petri dish (90 mm) by the third day with an abundant intertwined medium gray aerial mycelium turned to dark gray with a beige reverse (Fig. 1c, d). On CYA medium, NRCC6 colonies were sparse to cottony, dim gray when young turning fastly to brownish gray, reached 90 mm in diameter on the fifth day along with light brown reverse (Fig. 1e, f). In line with our results, Jakovljević and Vrvić (2017) suggested that genus *Mucor* were cotton candy colonies that grow rapidly at 25–30 °C, dark grayish-brown or light olive-gray aerial mycelium when grown on typical laboratory media.

Moreover, the asexual morph of NRCC6 based on cultures grown in MEA at 25 °C in Fig. 1g–l showed hyaline, erect, circinated, tall needle-like (up to 2 cm) and branched sporangiophores that were born from aerial hyphae and bearing large sporangia about 200–300 μm, globose, slightly flattened, brown, and multispored with encrusted walls become deliquescent and rupture at maturity, but columellae are globular to ellipsoid, and conspicuous collarettes were noticeable at the base of the columella following sporangiospore dispersal which is hyaline, ellipsoidal, and smooth-walled. Lima et al. (2020) reported that morphological features commonly used for species-level delimitation are the size and shape of structures such as sporangiospores and columella. Several authors have suggested that *Mucor circinelloides* varies from other *Mucor* species in its formation of short circinated, branched sporangiophores bearing brown sporangia (De Hoog et al. 2000, 2015; Hibbett et al. 2007; Rayner 1970; Rippon 1988; Schipper 1976, 1978; Schipper and Samson 1994; Vellanki et al. 2018; Watanabe 2009).

Interestingly, in all media, strain NRCC6 was faintly aromatic, and the growth was restricted at 10–15 °C, good between 16 and 18 °C, and excellent growth with abundant sporulation at 20–25 °C, but no growth was observed at 5 °C or above 32 °C. Then, we can report that the strain under study NRCC6 is a nonpathogenic strain because it cannot grow or sporulate at a temperature above 32 °C. Then, NRCC6 strain is safe for handling and environmental applications. Thermotolerance in *Mucor* usually hints at pathogenic potential; however, the inability of *Mucor hiemalis* and *Mucor racemosus* to grow at temperatures above 32 °C raises doubt as to their validity as human pathogens and their pathogenic role (De Hoog et al. 2000, 2015). However, some groups of *Mucorales* show morphological plasticity; hence, their taxonomy cannot be based solely on morphological features as these might not always be taxonomically informative. Then, the strain was subjected to genotypic characterization.

#### Molecular identification

**Fig. 2:**
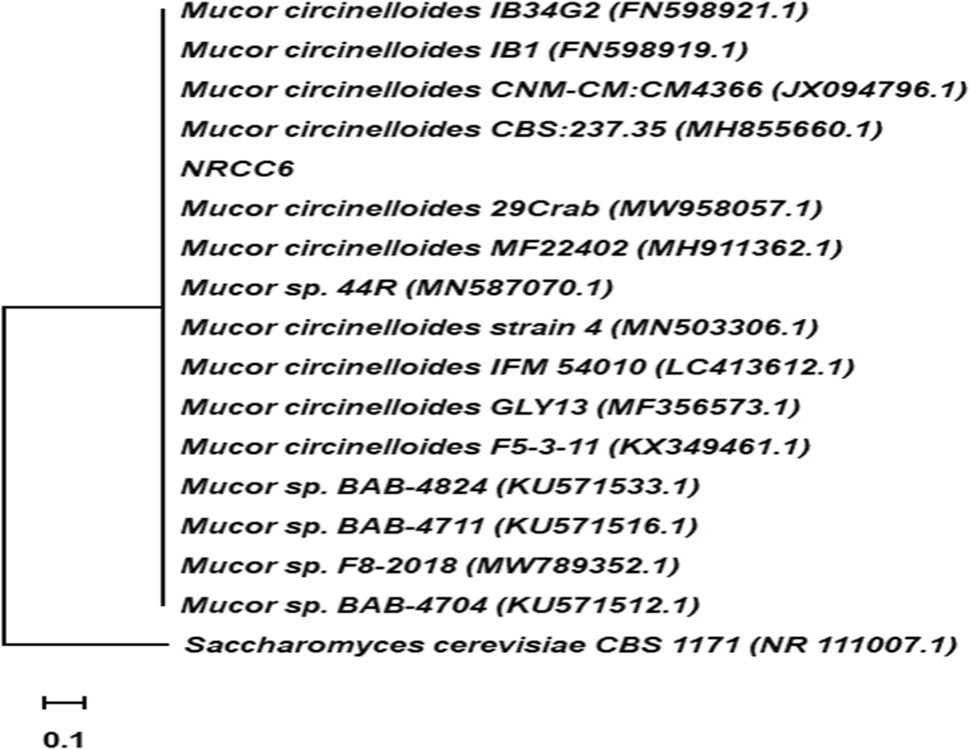
Phylogenetic tree generated by the neighbor-joining based method in the rDNA sequences of the ITS region with isolate NRCC6 belonging to the genus *Mucor* obtained through with 1000 repetitions. *Saccharomyces cerevisiae* was used as an outgroup

PCR product amplification of the ITS regions of the isolate NRCC6 strain generated 566 bp using ITS1 and ITS4 primers as well as submission to GenBank (accession number ON860507). Close examination revealed the characters commonly associated with the genus *Mucor*. The difference in the percentage of nucleotides in the ITS marker to its sister taxa is 0.53–2.47% with various strains in the genus *Mucor*. Therefore, phylogenetic analyzes were performed to obtain greater reliability in identification. Phylogenetics analysis by Blastn showed that the sequence of the ITS region of the NRCC6 strain has an important identity for several of the genus *Mucor*. Comparison of strain NRCC6 with the sequences of reference species in the bank database showed that strain NRCC6 has 99.47% similarity with *Mucor circinelloides* isolates 29 Crab, CBS 237.35, CNM-CM:CM4366, IB34G2, and IB1 (Fig. 2). Phylogenetic analyze and evolutionary history were performed by MEGA11 and neighbor-joining method (Tamura et al. 2021; Felsenstein 1985; Saitou and Nei 1987). According to the sequence analysis of the ITS region, along with its phenotypic characteristics, the NRCC6 isolate was identified as *Mucor* sp. and designated as *Mucor* sp. NRCC6. Most investigations focused on the identification of *Mucor* species that use ITS and LSU because these are the most widely available genetic marker (Chai et al. 2019; Lima et al. 2020). Recently, genetics approaches using hundreds of genes have begun to emerge to identify problematic fungal taxa (Vandepol et al. 2020). Previously, the fungal strains NRCF5, Gen 9, Gen 20, and ALAA-20 were identified as *Aspergillus* sp. NRCF5, *Trichoderma* sp. Gen 9, *Cladosporium* sp. Gen 20, and *Fusarium* sp. ALAA-20, respectively, based on their phenotypic and chemotypic characteristics combined with ITS sequence analysis (El-Bondkly 2012; El-Gendy et al. 2017b; El-Bondkly et al. 2021).

### Optimization of operating conditions for different heavy metal removal by *Mucor* sp. NRCC6 in multimetal aqueous solution using batch experiments

**Fig. 3:**
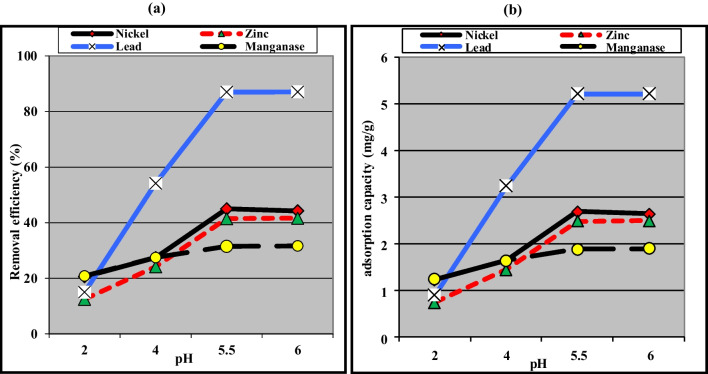
Influence of pH on the removal (**a**) and adsorption (**b**) capacity of different heavy metals by the dead biomass of *Mucor* sp. NRCC6

pH plays a critical role in heavy metal removal by fungi since it affects the speciation of the metals in solution and the surface properties of the fungi (El-Gendy et al. 2017a). As displayed in Fig. 3, the increase in the initial pH from pH 2.0 to pH 6.0 resulted in a significant improvement in the removal efficiency of Pb^2+^, Zn^2+^, and Mn^2+^ from 15.0, 12.5, and 20.83% to 87.08, 41.67, and 31.67% along with increasing the adsorption capacity to 5.23, 2.49, and 1.90 mg/g. These data can be attributed to the fact that at an acidic pH (pH 2.0–4.0), the adsorption sites of NRCC6 became saturated with a positively charged hydrogen ion (H^+^) which could compete with Pb^2+^, Ni^2+^, Mn^2+^, and Zn^2+^ ions for the active locations, but as the pH of the solution increases, the negatively charged OH tends to dominate the adsorption sites (El-Gendy and El-Bondkly 2016). This trend enhanced the electrostatic attraction between the Pb^2+^, Ni^2+^, Mn^2+^, and Zn^2+^ ions, and the -ve sites, thus enhancing their removal efficacy (Fei and Hu 2022). Overall, the highest removal (45.00%) and adsorption (2.70 mg/g) of Ni^2+^ were detected on NRCC6 biomass at pH 5.5 (Fig. 3). Our data are in agreement with those of Zhang et al. (2020) in which low pH led to a negative effect on adsorption performance because it could accelerate the dissolution/oxidation of functional groups on the adsorbent surface, leading to ion release, and desorption of entrapped heavy metals.

As depicted in Table 2, contact time of 30 min supported the highest removal efficiency (33.25, 46.33, and 87.50%) and adsorption capacity (1.99, 2.78, and 5.25 mg/g) of Mn^2+^, Ni^2+^, and Pb^2+^, respectively. The fast adsorption capacity through the initial stage was probably because of the abundance of vacant active sites on the biomass and the high concentration gradient of solutes Pb^2+^, Ni^2+^, and Mn^2+^ in the multimetal solution (Alzahrani et al. 2017; Alzahrani and El-Gendy 2019). However, the removal and adsorption rates of Mn^2+^, Ni^2+^, and Pb^2+^ ions were decreased to reach 29.17% and 1.75 mg/g, 45.83% and 2.75 mg/g, and 87.17% and 5.23 mg/g, respectively, with increasing contact time to 180 min (Table 2). On the other hand, the removal and adsorption of Zn^2+^ were slower under the multi-adsorption condition and reached its maximum values (42.92% and 2.58 mg/g) at contact time of 90 min (Table 2). The decreasements in removal and adsorption capacity during later stages may be attributed to the agglomeration of Pb^2+^, Ni^2+^, and Mn^2+^ onto the *Mucor* sp. NRCC6 active sites. The difficulty in occupying the remaining binding sites can be caused by the forces between the solute molecules in the solid and bulk phases and the eversible interaction; hence, these ions diffuse successfully from the boundary layer surrounding the NRCC6 molecules into the bulk solution as previously reported (Alothman et al. 2020; Chen et al. 2019; Gola et al. 2018; Kumar et al. 2022).

**Table 2:** Influence of contact time on the bioremoval efficiency (%) and adsorption capacity (mg/g) of metal ions by the dead biomass of the fungus *Mucor* sp. NRCC6

Contact time (min)	Metal ion							
	Mn^2+^		Ni^2+^		Pb^2+^		Zn^2+^	
	Removal (%)	Adsorption (mg/g)	Removal (%)	Adsorption (mg/g)	Removal (%)	Adsorption (mg/g)	Removal (%)	Adsorption (mg/g)
10	22.50	1.35	31.67	1.90	62.00	3.72	18.33	1.10
20	29.17	1.75	40.75	2.45	80.42	4.83	27.50	1.65
30	33.25	1.99	46.33	2.78	87.50	5.25	41.58	2.49
60	31.50	1.89	45.00	2.70	87.50	5.25	41.46	2.48
90	30.00	1.80	45.50	2.73	87.08	5.23	42.92	2.58
180	29.17	1.75	45.83	2.75	87.17	5.23	42.92	2.58

In this study, it was observed that the equilibrium between the sorbent NRCC6 biomass and the sorbates Mn^2+^ and Zn^2+^ in the multimetal solution was achieved after increasing the biomass dose to 10.0 g/L, as the bioremoval efficiency reached their maximum values (58.33 and 50.83%), respectively (Table 3). This data could be attributed to the availability of more vacant sites concerning the metal ions at higher NRCC6 dosage, but the increase in removal at initial biosorbent doses could be attributed to the greater surface area of the biosorbent, which in turn increased the availability of the active sites for metal ions (Ayele et al. 2021; El-Gendy et al. 2011). Furthermore, *Mucor* sp. NRCC6 biomass at a concentration of 5.0 g/L supported the best removal of Ni^2+^ and Pb^2+^ (59.25 and 98.75%, respectively) from the multimetal solution and then decreased at a higher biomass dose (Table 3). Kucuker et al. (2017) stated that the reduction of biosorption efficiency at a high biomass dosage can be attributed to the shell effect mechanism that protects the active biosorbent binding sites and stops the metal ions occupying a proportion of the sites. However, the highest adsorption capacity of Mn^2+^, Ni^2+^, Pb^2+^, and Zn^2+^ ions from the multimetal solution into the *Mucor* sp. NRCC6 biomass (3.0, 5.0, 9.3, and 4.0 mg/g) was achieved at 1.0 g/L biomass dose (Table 3). In the case of higher biosorbent concentrations, the decreased adsorption due to excess of the adsorbent dose could cause an overlap of the pore structure of the adsorbent and increase the screening effect of the boundary layer, which shields binding sites from metals and reduces interparticle distances (Kumar et al. 2019; Sharma et al. 2022).

**Table 3:** Effect of biosorbent dose on the bioremoval efficiency (%) and adsorption capacity (mg/g) of metal ions by the dead biomass of the fungus *Mucor* sp. NRCC6

Dose (g/L)	Metal ion							
	Mn^2+^		Ni^2+^		Pb^2+^		Zn^2+^	
	Removal (%)	Adsorption (mg/g)	Removal (%)	Adsorption (mg/g)	Removal (%)	Adsorption (mg/g)	Removal (%)	Adsorption (mg/g)
1	25.00	3.00	41.67	5.00	77.50	9.30	33.33	4.00
2	33.25	1.99	46.33	2.78	87.50	5.25	41.58	2.49
5	57.08	1.37	59.25	1.42	98.75	2.37	49.17	1.18
10	58.33	0.70	57.50	0.69	97.08	1.17	50.83	0.61

The initial metal concentration is an important factor affecting the biosorption process. Active binding sites and functional groups, available on the biosorbent surface, are affected by the initial concentration of metal ions (Tu et al. 2018). The highest removal capacities for Pb^2+^, Ni^2+^, Mn^2+^, and Zn^2+^ were estimated to be 98.75, 59.25, 57.08, and 49.17%, respectively at the initial concentration of 12 mg/L per metal ion in the multimetal aqueous solutions, respectively (Table 4). The adsorption improvement in the initial stages can be attributed to the greater driving force of metal ions on the fungal surface and the abundance of vacant binding sites on the biosorbent surface (Alzahrani and El-Gendy 2019; Kumar et al. 2019). Data in Table 4 showed that the multi-sorption of Pb^2+^, Ni^2+^, Zn^2+^, and Mn^2+^ by NRCC6 biomass from the solution was significantly reduced at the initial concentration ˃ 12 mg/L per metal in the mixture (Table 4). This observation was due to the lack of active binding sites available on the surface of the biosorbent at higher metal concentrations (Satya et al. 2020). The removal capacity of Pb^2+^, Ni^2+^, Mn^2+^, and Zn^2+^ decreased to 92.0, 54.0, 52.0, and 34.0% at 25.0 mg/L; 84.0, 52.0, 34.0, and 20.0% at 50 mg/L; and 75.0, 45.0, 25.0, and 14.2% at 100 mg/L of each ion, respectively, in the aqueous solutions (Table 4). Similarly, Tu et al. (2018) reported that the decrease in removal of heavy metals at higher concentrations of metals could be related to the excess amount of ions with respect to saturation of all available sorption locations on the fungal surface and equilibrating the sorbent and sorbate, thus preventing further adsorption of metal ions.

**Table 4:** Effect of initial metal concentrations on the bioremoval efficiency (%) and adsorption capacity (mg/g) of metal ions by the dead biomass of the fungus *Mucor* sp. NRCC6

Initial conc. (mg/L)	Metal ions															
	Mn^2+^				Ni^2+^				Pb^2+^				Zn^2+^			
	Removal (%)	Residual conc. (mg/L)	Adsorption conc. (mg/L)	Adsorption capacity (mg/g)	Removal (%)	Residual conc. (mg/L)	Adsorption conc. (mg/L)	Adsorption capacity (mg/g)	Removal (%)	Residual conc. (mg/L)	Adsorption conc. (mg/L)	Adsorption capacity (mg/g)	Removal (%)	Residual conc. (mg/L)	Adsorption conc. (mg/L)	Adsorption capacity (mg/g)
12	57.08	5.15	6.85	1.37	59.25	4.89	7.11	1.42	98.75	0.15	11.85	2.37	49.17	6.10	5.90	1.18
25	52.00	12.00	13.00	2.60	54.00	11.50	13.50	2.70	92.00	2.00	23.00	4.60	34.00	16.50	8.50	1.70
50	34.00	33.00	17.00	3.40	52.00	24.00	26.00	5.20	84.00	8.00	42.00	8.40	20.00	40.0	10.00	2.00
100	25.00	75.00	25.00	5.00	45.00	55.00	45.00	9.00	75.00	25.00	75.00	15.00	14.20	85.80	14.20	2.84

On the contrary, the adsorption capacity of Pb^2+^, Ni^2+^, Zn^2+^, and Mn^2+^ was increased by NRCC6 as the initial concentration of each metal was increased until it reached its maximum values of 15.0, 9.0, 5.0, and 2.84 mg/g, respectively, at a concentration of 100 mg/L of each metal ions in the multimetal solution (Table 4). The initial metal ion concentration strongly affects the metal adsorption process, as it provides an important driving force to overcome all mass transfer resistances of metal ions between the aqueous and solid phases (Velkova et al. 2018). Zhang et al. (2017) reported that *M. circinelloides* selected from mine tailings for heavy metal bioremediation could adsorb 79.5, 44.1, 62.5, 56.5, and 85.5% of Fe(III), Mn(II), Cu(II), Zn(II), and Pb(II), respectively, from an initial concentration of 20 mg/L under optimal conditions including pH 8.0 and 30 °C.

### Evaluation of adsorption isotherm models

Analyzing the data by adsorption equilibrium isotherms is important for proposing an adsorption system. In the present work, the adsorption data between the adsorbate ions Pb^2+^, Ni^2+^, Mn^2+^, and Zn^2+^ and dead fungal biomass of NRCC6 (an adsorbent) at equilibrium were evaluated by different adsorption isotherms including Langmuir, Freundlich, and Dubinin–Kaganer–Radushkevich (DKR) models.

**Fig. 4:**
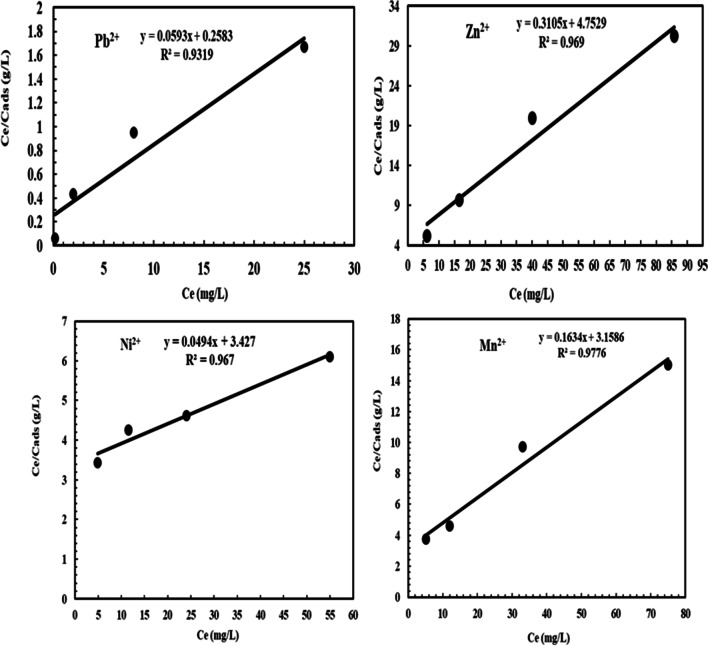
Langmuir isotherm model for Pb^2+^, Zn^2+^, Ni^2+^, and Mn^2+^ adsorption onto NRCC6 dead biomass

The Langmuir isotherm model for Pb^2+^, Ni^2+^, Mn^2+^, and Zn^2+^ adsorption on NRCC6 showed that *Q*_max_ = 16.86, 20.25, 6.12, and 3.22 mg/g as well as *K* = 47.57, 0.846, 2.84, and 4.15 L/mg, respectively (Table 5 and Fig. 4). Furthermore, the determination of the coefficient of the linear equation for Langmuir was *R*^2^ = 0.9319, 0.967, 0.9776, and 0.969 for Pb^2+^, Ni^2+^, Mn^2+^, and Zn^2+^, respectively (Table 5 and Fig. 4). This observation implied that the experimental inputs pH, temperature, biomass dosage, contact time, and initial metal ion concentration showed positive and linear impacts on the model output beside no over-fitting problem occurred during prediction. The Langmuir model could adequately describe the adsorption mechanism of Pb^2+^, Zn^2+^, Ni^2+^, and Mn^2+^ onto fungal biomass NRCC6 at equilibrium. The RL values lie in the range 0 < RL < 1 indicating that the adsorption of these heavy metal ions on surface NRCC6 is favorable, and then, the Langmuir model gives a good fit to the adsorption process, which was established from the positive values gotten for the Langmuir constants presented in Table 6. Khayyun and Mseer (2019) reported that the adsorption is favorable at 0 < RL < 1, irreversible at RL = 0, linear at RL = 1, and unfavorable RL > 1.

**Table 5:** Summary of isotherm model parameters for Ni^2+^, Pb^2+^, Mn^2+^, and Zn^2+^ ion adsorption on *Mucor* sp. NRCC6 dead biomass

Ion adsorption	Langmuir			Freundlich			Dubinin–Kaganer–Radushkevich (DKR)			
	KL L/mg	*q*_max_ (mg/g)	*R*^2^	*K*_*f*_	*n*	*R*^2^	Xm (mol/g)	β (mol^2^/j^2^)	E, KJ/mol	*R*^2^
Pb^2+^	47.57	16.86	0.9319	3.282	2.14	0.9979	2.04 × 10^−4^	0.2438 × 10^−8^	14.32	0.9471
Zn^2+^	4.15	3.22	0.969	0.681	3.3	0.9184	9.18 × 10^−5^	0.3064 × 10^−8^	12.774	0.9006
Ni^2+^	0.846	20.25	0.967	0.419	1.29	0.9972	1.52 × 10^−3^	0.769 × 10^−8^	8.065	0.9975
Mn^2+^	2.84	6.12	0.9776	0.714	2.19	0.9594	3.22 × 10^−4^	0.4705 × 10^−8^	10.309	0.9695

**Table 6:** Analysis of detergent industrial effluent before and after adsorption by *Mucor* sp. NRCC6 dead biomass compared to FEPA/WHO standards

*Parameter	Non-treated	Treated	Reduction values (%)	FEPA/WHO standards
Temp (°C)	25.5 ± 0.0	30.0 ± 0.0	−17.65	40
Turbidity (NTU)	21.41 ± 2.16	5.00 ± 0.47	76.65	NS
pH	2.0 ± 0.01	5.91 ± 0.03	−195.50	6–9
TSS	3600 ± 41.15	715.99 ± 14.25	80.11	30
TDS	10000 ± 80.47	2024.00 ± 17.30	79.76	NS
Oil and grease	282 ± 15.14	10.16 ± 1.19	96.40	NS
COD	4687 ± 43.40	1038.00 ± 18.44	77.85	200
BOD	3200 ± 34.27	990.13 ± 18.76	69.06	30
Dissolved oxygen (DO)	6.43 ± 0.45	1.91 ± 0.34	70.30	>2
Cr^6+^	5.70 ± 0.51	2.28 ± 0.32	60.00	0.05
Cd^2+^	0.19 ± 0.04	0.04 ± 0.0	78.95	0.003
As^2+^	0.35 ± 0.08	0.11 ± 0.0	68.57	0.01
Cu^2+^	1.50 ± 0.21	0.39 ± 0.04	74.00	2.0
Pb^2+^	0.10 ± 0.02	0.0 ± 0.0	100.0	0.01
Ni^2+^	2.30 ± 0.30	0.06 ± 0.0	97.39	0.07
Mn^2+^	40.80 ± 3.62	4.61 ± 0.46	88,70	NS
Zn^2+^	19.0 ± 1.05	0.00 ± 0.0	100.0	NS
Fe^3+^	410.80 ± 19.21	122.33 ± 8.52	70.22	NS
PO4^3−^	91.56 ± 7.81	22.14 ± 2.39	75.82	5
SO_4_^2−^	411.20 ± 22.54	115.78 ± 7.11	71.84	500
NO_3_^−1^	26.29 ± 2.01	4.77 ± 0.48	81.86	20
NH_4_–N	193.5 ± 12.44	36.21 ± 3.60	81.29	15

*NS*, not specified. *All determents in mg/L, pH in pH units, temperature in °C, and turbidity in NTU. NM means not mentioned

**Fig. 5:**
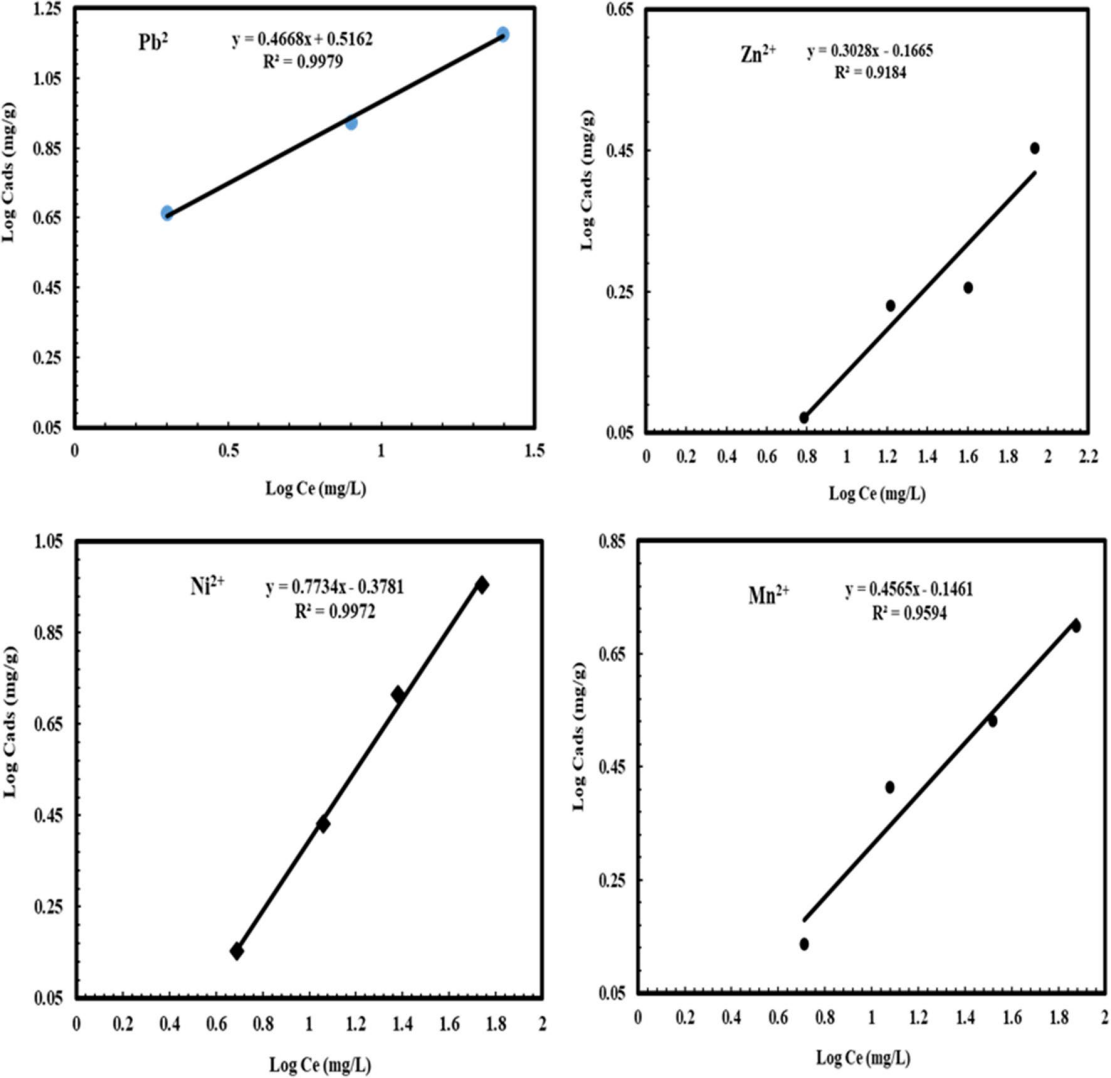
Freundlich isotherm model for Pb^2+^, Zn^2+^, Ni^2+^, and Mn^2+^ adsorption onto NRCC6 dead biomass

From the parameters of the Freundlich isotherms listed in Table 5 and Fig. 5, *n* values are 2.14, 1.29, 2.19, and 3.3; *K*_*f*_ = 3.282, 0.419, 0.714, and 0.681; and *R*^2^ = 0.9979, 0.9972, 0.9594, and 0.9184 for Pb^2+^, Ni^2+^, Mn^2+^, and Zn^2+^, respectively. El-Gendy et al. (2017a) reported that values of *K*_*f*_ and *n* determine the steepness, the isothermal curvature, and the adsorption capacity of the adsorbent that increase with a higher value of *K*_*f*_. Hence, the adsorption capability of NRCC6 adsorbent was in the order of Pb^2+^ (3.282) ˃ Mn^2+^ (0.714) ˃ Zn^2+^ (0.681) ˃ Ni^2+^ (0.419), respectively (Table 5). On the other hand, Khayyun and Mseer (2019) and El-Gendy et al. (2011) reported that the adsorption process is desirable when 0.1< 1/*n* < 1, irreversible at 1/*n* = 0, and unfavorable at 1/n > 1. Hence, the 1/*n* values of 0.467, 0.775, 0.457, and 0.303 for Pb^2+^, Ni^2+^, Zn^2+^, and Mn^2+^, respectively, suggested that the isotherm type is desirable, favorable, and refer to the strong interaction between fungal biomass and these heavy metals under multimetal sorption condition (Table 6). Apart from a homogeneous surface, the Freundlich equation is also suitable for a highly heterogeneous surface and an adsorption isotherm lacking a plateau, indicating multi-layer adsorption (Azizian and Eris 2021; Sayed et al. 2019).

**Fig. 6:**
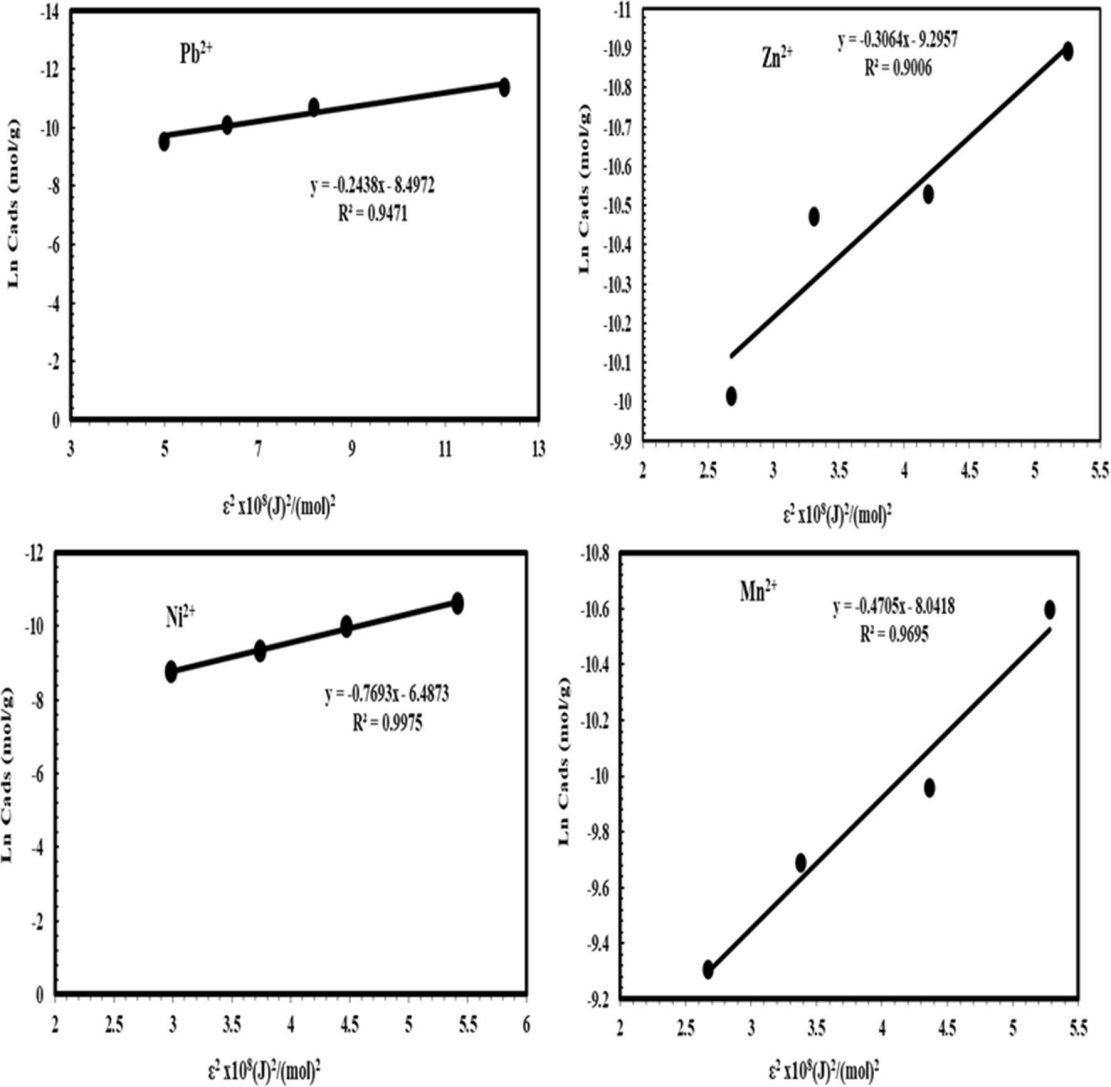
Dubinin–Kaganer–Radushkevich (DKR) isotherm model for Pb^2+^, Zn^2+^, Ni^2+^, and Mn^2+^ adsorption onto NRCC6 dead biomass

The DKR isotherm model in Table 5 and Fig. 6 was selected to assess the characteristic porosity of the biomass and the apparent energy of adsorption as well as describe the equilibrium between the adsorbates Pb^2+^, Ni^2+^, Mn^2+^, and Zn^2+^ and the adsorbent NRCC6. The maximum sorption capacity (saturation capacity in mol/g), values, and *X*_*m*_ describing the total specific micropore volume of the sorbent were estimated to be 2.04 × 10^−4^, 1.52 × 10^−3^, 3.22 × 10^−4^, and 9.18 × 10^−5^ mol/g for Pb^2+^, Ni^2+^, Mn^2+^, and Zn^2+^, respectively (Table 5). Furthermore, the parameters of the DKR model listed in Table 5 indicated that the determination of the linear equation coefficient for DKR was *R*^2^ = 0.9471, 0.9975, 0.9695, and 0.9006 for Pb^2+^, Ni^2+^, Mn^2+^, and Zn^2+^, respectively (Table 5). The positive values of the free energy *E* estimated for Pb^2+^, Ni^2+^, Mn^2+^, and Zn^2^ (14.32, 8.065, 10.309, and 12.774 KJ/mol) indicated the endothermic nature of the sorption process of these heavy metal ions by NRCC6 dead biomass, respectively (Table 5). The adsorption nature was defined based on the *E* values as physical adsorption (*E* < 8 kJ/mol), ion exchange (*E* = 8-16 kJ/mol), and stronger chemical adsorption (*E* ˃16 kJ/mol). Then, based on the energy values, the sorption process onto NRCC6 can be construed by ion exchange. Moreover, comparing the *R*^2^ values in the Langmuir (0.9319, 0.967, 0.9776, and 0.969), Freundlich (0.9979, 0.9972, 0.9594, and 0.9184), and DKR (0.9471; 0.9975, 0.9695, and 0.9006) isotherm models for the adsorption of Pb^2+^, Ni^2+^, Mn^2+^, and Zn^2+^, respectively, onto NRCC6 could indicate that Langmuir isotherm is the best for describing the adsorption of Zn^2+^ (*R*^2^= 0.969) and Mn^2+^ (*R*^2^= 0.9776) and Freundlich isotherm significantly giving a good fit to the adsorption of Pb^2+^ (*R*^2^= 0.9979) while the sorption of Ni^2+^ onto NRCC6 biomass can follow DKR isotherm (*R*^2^= 0.9975) followed by Freundlich (*R*^2^= 0.9972) (Table 5). Previously, Igwe and Abia (2007) reported that depending on the *R*^2^ values of three isotherm models, Dubinin–Radushkevich is the best followed by Freundlich, but Langmuir does not give a good fit to the sorption process of Pb(II) and Zn(II) ions from wastewater.

### Effect of Pb^2+^, Ni^2+^, Mn^2+^, and Zn^2+^ blind on NRCC6 mycelial surface morphology by SEM, EDX, and FTIR spectroscopy

**Fig. 7:**
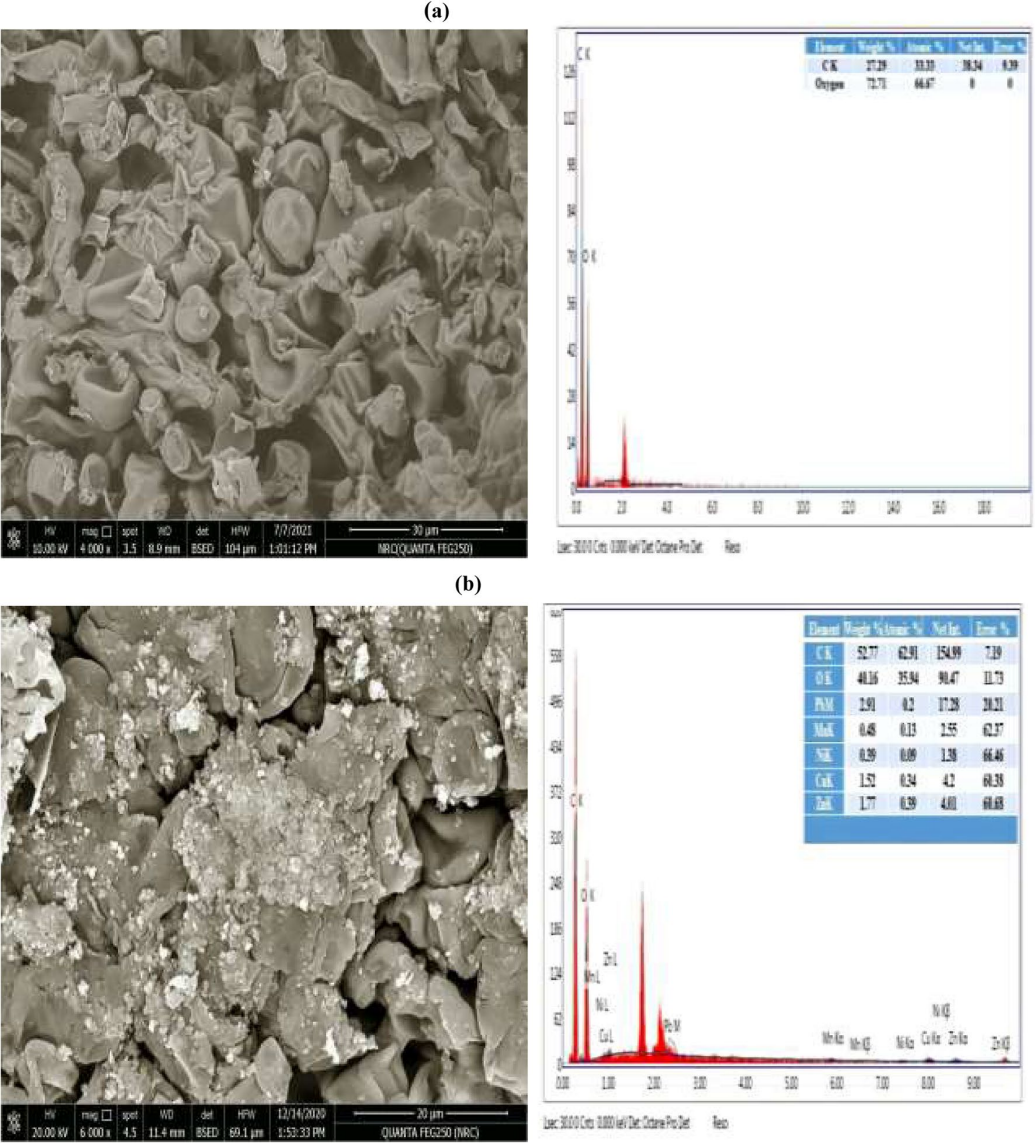
The SEM and EDX analysis of the surface morphology of *Mucor* sp. NRCC6 dead biomass before (**a**) and after (**b**) adsorption at the optimal operating conditions (pH; 6.0 for Pb^2+^, Zn^2+^, Mn^2+^, and 5.5 for Ni^2+^, Tem; 30 °C, agitation speed; 150 rpm, contact time; 30 min for Mn^2+^, Ni^2+^, Pb^2+^, and 90 min for Zn^2+^ and adsorbent dosage; 5.0 g/L for Ni^2+^, Pb^2+^, and 10.0 g/L for Mn^2+^, Zn^2+^, and initial metal concentration; 12 mg/L of each ion in the multimetal aqueous solution)

The control (unloaded) biomass of *Mucor* sp. NRCC6 showed distinct and regular hyphal shape, an intact long rod, cylindrical sheets, or even ribbon-shaped mycelial fibers that were branched and intertwined as well as a small number of uniform oval or conical-like structures that were observed with no visible physical damage (Fig. 7a). On the contrary, SEM images of NRCC6 biomass after metal ion blend adsorption showed blocking of the vacant sites, indicating the attachment of metal ions onto the NRCC6 surface along with broad mycelium deformations (Fig. 7b). Furthermore, changes in hyphal shape that developed closely to form a large expansion of irregular thick mycelial mass in addition to irregular expansion distorted and damaged with significant visual abnormalities in the treated NRCC6 mycelium were observed (Fig. 7b). Noticeable amounts of Pb^2+^, Ni^2+^, Mn^2+^, and Zn^2+^ were also detected to be adsorbed on the fungal mycelia after the treatment with metal ion blend (Fig. 7b). Morphological variations in the fungal mycelia under heavy metal stress have been described earlier, which might be because of the oxidation of protein and DNA molecules, alterations in ultrastructure, or inhibition of antioxidant defense system in cell, and the degree of damage might vary with different fungal strains depending on their ability to overcome metal stress conditions (Liaquat et al. 2020). These changes after the bioremediation process are attributed to the adsorption and accumulation of toxic metal ions resulting in an increased area for the interaction of metal ions that cause alterations at physiological, morphological, cellular, and molecular levels (El-Gendy et al. 2011, 2017a).

However, EDX spectra in Fig. 7a showed detectable peaks of C (27.29%) and O (72.71%) in the unloaded biomass of *Mucor* sp. NRCC6 while detectable peaks of Pb^2+^ (2.91%) and Zn^2+^ (1.77%) with smaller peaks of Mn^2+^ (0.48%) and Ni^2+^ (0.39%) ions have been observed after bioremediation in the multimetal-treated fungus EDX spectra (Fig. 7b). These values of Pb^2+^, Zn^2+^, Ni^2+^, and Mn^2+^ ion adsorption suggested intracellular accumulation of these metals rather than binding on the cell surface. Similarly, laser scanning microscopy images of the wood-rot fungus *Schizophyllum commune* demonstrated an intracellular metal accumulation primarily within vacuoles or vesicles, and the maximum adsorption amount was detected in the apical cells along with the swelling of the hyphal tip (Traxler et al. 2022). Interestingly, compared to the untreated NRCC6 sample (Fig. 7a, b), the amount of O(II) decreased from 72.71 to 40.16% while the amount of C increased from 27.29 to 52.77% in the multimetal-loaded sample. In line with our results, Majlesi and Hashempour (2017) reported that the appearance of a peak at the energy level of Kα = 0.5 keV, Lα = 0.93 keV, and Mα= 8.91 keV deduced the successfulness of metal ion adsorption onto the fungal surface. Furthermore, Hoque and Fritscher (2019) stated that SEM-EDX analysis revealed the binding and precipitation of metal ions as spherical nano-particles (~50–100 nm) at the outer electro-negative cell wall-surface of *Mucor* sp. EH8, EH10, and EH11 sporangiospores.

**Fig. 8:**
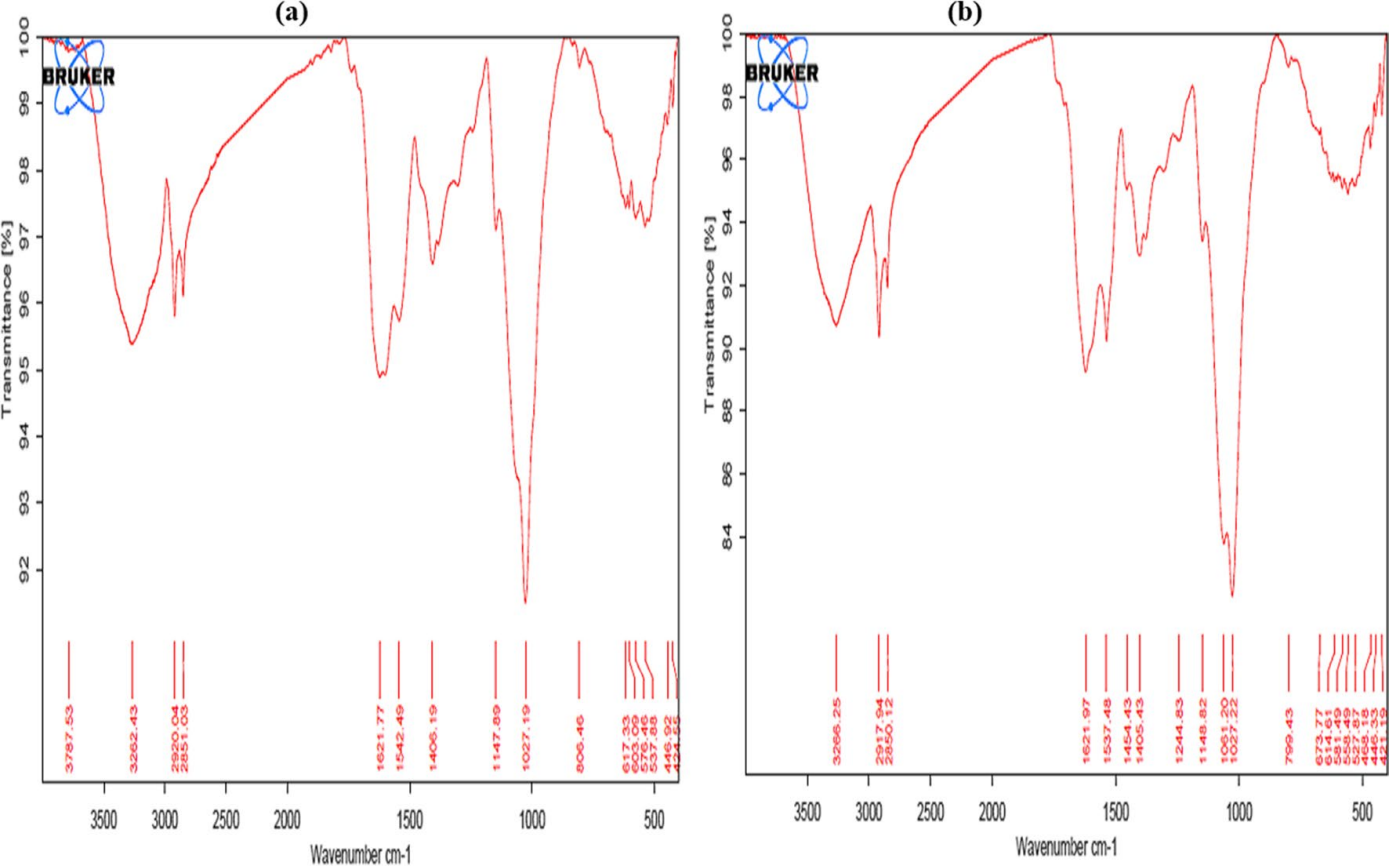
The FTIR analysis of the unloaded (**a**) and loaded (**b**) *Mucor* sp. NRCC6 dead biomass before and after adsorption

The FTIR spectra of NRCC6 before and after Pb^2+^, Ni^2+^, Mn^2+^, and Zn^2+^ adsorption experiments are presented in Fig. 8a, b. Distinctive beaks in the unloaded-NRCC6 dead biomass at 3262.43, 2920.04, 2851.03, 1621.77, 1542.49, 1406.19, 1147.89, 1027.19, 806.46, 617.33, 576.46, 537.88, 446.92, and 424.55 cm^−1^ before the adsorption process were detected (Fig. 8a). After adsorption, these peaks were shifted to 3266.25 cm^−1^ (strong ≡C-H stretch), 2917.94–2850.12 cm^−1^ (weak -C-H stretch), 1621.97–1537.48 cm^−1^ (strong C=O amide), 1405.43 cm^−1^ (weak C=C aromatic), 1148.82 cm^−1^ (strong C-O stretching), 1027.22 cm^−1^ (strong C-O stretching), 799.43 cm^−1^ (medium C=C bending), 614.61, 581.49, and 527.87 cm^−1^ (strong C-Br stretching), and 446.33–421.19 cm^−1^ (C-I strong stretching), respectively (Fig. 8b). Compared to the untreated biomass, new characteristic peaks were created in the NRCC6-loaded biomass at 1454.43, 1244.83, 1061.20, 673.77, 558.49, and 468.18 cm^−1^ (Fig. 8b), which refer to the creation of medium C-H bending of alkane, medium C-N stretching of amine, strong S=O stretching of sulfoxide, strong C=C bending of alkene, strong C-Br stretching, and strong C-I stretching of halo compound, respectively, that can be created to bind with heavy metal ions (Fig. 8b). Moreover, the two characteristic peaks at 3787.53 cm^−1^ (refer to strong water OH) and 603.09 cm^−1^ (refer to stretch and strong C-Cl) detected in the control biomass were not detected after treatment (Fig. 8a, b). In line with our results, the characteristic infrared peaks before and after bioremoval reported in diverse literatures showed the involvement of fungal functional groups including hydroxyl, ethers, amines/amides, carboxylic acid, carbonyl, sulfhydryl, and phosphoryl groups in the adsorption of Cd^2+^ by *P. chrysosporium* (Noormohamadi et al. 2019), metal mix (Cu^2+^, Cr^6+^, Cd^2+^, Pb^2+^, and Zn^2+^) adsorption by *T. brevicompactum* QYCD-6 (Zhang et al. 2020), Cr^6+^ and Pb^2+^ adsorption by *P. chrysogenum* CS1 (Qian et al. 2017), and Pb^2+^ adsorption by *Pleurotus ostreatus* ISS-1 (Wang et al. 2019).

### Application of biosorption for real wastewater treatment

The data in Table 6 showed that the biomass of the fungal strain NRCC6 under the optimized process parameters evaluated above was an appropriate material for bioremediation of real industrial wastewater under coexistence of different metallic ions and other pollutants. NRCC6 dead biomass showed the highest removal efficiency (RE) toward Pb^2+^ and Zn^2+^ (100%) ˃ Ni^2+^ (97.39%) ˃ Mn^2+^ (88.70%) ˃ Cd^2+^ (78.95%) ˃ Cu^2+^ (74.00%) ˃ Fe^3+^ (70.22%) ˃ As^2+^ (68.57%) ˃ Cr^6+^ (60.00%) from industrial wastewater (Table 6). The perspective of filamentous fungi in removing of commercial detergent has been continuously described over the past three decades from the species of *Aspergillus*, *Penicillium*, *Rhizopus*, *Mucor*, *Humicola*, *Thermoascus*, and *Thermomyces* (Jakovljević and Vrvić 2017). In accordance with our results, Sharma et al. (2022) stated that using fungi including *P. chrysosporium*, *Phlebia brevispora*, and *Phlebia floridensis* is a new efficient and eco-friendly method that revealed a maximum removal of 98–99% for Ni, 97–98% for Cd, and 12–98% for Pb from the industrial wastewater. Overall, the results presented in Table 6 showed that removal values of 75.82, 71.84, 81.86, 81.29, 80.11, 79.76, 96.40, 77.85, 69.06, 70.30, and 76.65% were achieved for PO4^3−^, SO_4_^2−^, NO_3_^−1^, NH_4_–N, TSS, TDS, oil and grease, COD, BOD, DO, and turbidity from detergent industry effluent. Matched to our data, Ankesh et al. (2019) reported that treatment of wastewater from soap industries resulted in the removal of color, nitrate, fluoride, oil and grease, COD, BOD, total dissolved solids, chromium, electrical conductivity, and total suspended solids by 46.15, 27.27, 50.0, 65.21, 77.56, 63.84, 19.72, 56.81, 27.38, and 71.15%, respectively. Interestingly, the wastewater under study was highly acidic pH = 2.0 ± 0.01 which could be attributed to 1000, 59.6, 8.37, and 94.65 mg/L sulphate, sulphide, nitrate, and phosphate, respectively, previously detected and reported by Aonghusa and Gray (2002) in 93% of the detergent wastewater samples. After treating the effluent of the detergent industry under study with NRCC6 biomass, the pH value was increased from 2.0 ± 0.01 to 5.91 ± 0.03 (Table 6). Hoque and Fritscher (2019) reported that strains EH8, EH10, and EH11 strains of *Mucor hiemalis* in the microbiomes of several sulfur springs exhibited multimetal-resistance, excessive accumulation, and multimetal remediation for simultaneous removal, fractionation, and enrichment of metal ions.

## Conclusion

To find a new strategy for the bioremediation of metal ions from detergent industrial wastewater, the fungal microbiome of the detergent industry effluent was investigated for the biosorption capacity of the most harmful ions Mn^2+^, Zn^2+^, Ni^2+^ and Pb^2+^. Out of them, the fungus *Mucor* sp. NRCC6 exhibited the highest adsorption potential of multiple heavy metals from the polluted effluents including Pb^2+^, Ni^2+^, Zn^2+^, and Mn^2+^ because it adapted to the high concentrations of various pollutants in such harsh environmental conditions. The biosorption process of Zn^2+^ and Mn^2+^ onto dead NRCC6 biomass under multimetal system follows well the Langmuir isotherm while Freundlich isotherm was the best to describe the sorption of Pb^2+^ and Ni^2+^ onto NRCC6 biomass. Moreover, the cellular changes of the fungus induced by the multi-adsorption and intracellular accumulation of these heavy metals were explored by SEM-EDX and FTIR analysis to understand the mycoremediation process to remove pollutants from the environment more efficiently and quickly. Based on the data obtained, it can be concluded that indigenous fungi can be applied as a natural economic strategy for effective mycoremediation of industrial effluents that contain a large amount of recalcitrant, persistent, and toxic heavy metals as well as prevent the deleterious effect on the environment and human health. Under optimized operating conditions, NRCC6 biomass has proven to be a suitable material handling the coexistence of various metal ions including Cr^6+^, Cd^2+^, As^2+^, Pb^2+^, Cu^2+^, Ni^2+^, Mn^2+^, Zn^2+^, and Fe^3+^in the industrial effluents. Moreover, it was able to reduce PO4^3−^, SO_4_^2−^, NO_3_^−1^, and NH_4_–N in addition to turbidity, TSS, TDS, COD, BOD, and oil and grease with high efficiency from industrial effluent.

## Data Availability

All data generated or analyzed during this study are included in this published article.
